# Structure of human drug transporters OATP1B1 and OATP1B3

**DOI:** 10.1038/s41467-023-41552-8

**Published:** 2023-09-18

**Authors:** Anca-Denise Ciută, Kamil Nosol, Julia Kowal, Somnath Mukherjee, Ana S. Ramírez, Bruno Stieger, Anthony A. Kossiakoff, Kaspar P. Locher

**Affiliations:** 1 Institute of Molecular Biology and Biophysics, Department of Biology, ETH Zürich, Zürich, Switzerland; 2 Department of Biochemistry and Molecular Biology, The University of Chicago, Chicago, IL USA

**Keywords:** Cryoelectron microscopy, Membrane proteins, Permeation and transport

## Abstract

The organic anion transporting polypeptides OATP1B1 and OATP1B3 are membrane proteins that mediate uptake of drugs into the liver for subsequent conjugation and biliary excretion, a key step in drug elimination from the human body. Polymorphic variants of these transporters can cause reduced drug clearance and adverse drug effects such as statin-induced rhabdomyolysis, and co-administration of OATP substrates can lead to damaging drug-drug interaction. Despite their clinical relevance in drug disposition and pharmacokinetics, the structure and mechanism of OATPs are unknown. Here we present cryo-EM structures of human OATP1B1 and OATP1B3 bound to synthetic Fab fragments and in functionally distinct states. A single estrone-3-sulfate molecule is bound in a pocket located in the C-terminal half of OATP1B1. The shape and chemical nature of the pocket rationalize the preference for diverse organic anions and allow in silico docking of statins. The structure of OATP1B3 is determined in a drug-free state but reveals a bicarbonate molecule bound to the conserved signature motif and a histidine residue that is prevalent in OATPs exhibiting pH-dependent activity.

## Abstract (web summary)

A key step of drug metabolism in the human body is the uptake into liver cells, which is mediated by transport proteins of the OATP family. Here, authors report cryo-EM structures of two human OATP proteins, providing insight into their function.

## Introduction

**Fig. 1:**
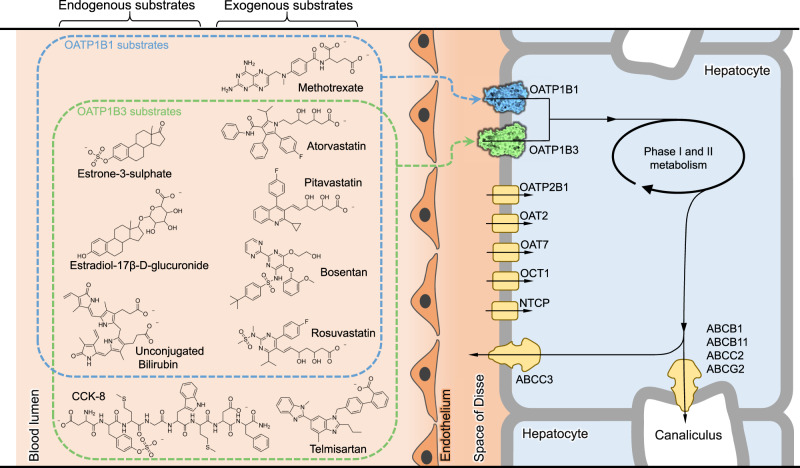
Physiological role of human OATP1B1 and OATP1B3. The schematic shows the uptake of endogenous and exogenous substrates from the blood lumen (systemic circulation) via the space of Disse into hepatocytes. Transporters in the basolateral membrane are shown as surfaces (OATP1B1 and OATP1B3) or yellow shapes. The chemical structures of a selection of substrates of OATP1B1 and/or OATP1B3 are shown on the left and are grouped by whether they are endogenous (left column) or exogenous (right column). The chemical structures were prepared using ChemDraw.

The liver facilitates vital processes due to its strategic location between the gut and the systemic circulation. It is exposed to most nutrients and xenobiotics, and consequently has a central role in protecting the body from toxic compounds by metabolism and excretion. These processes require the uptake of substances and their metabolites across the plasma membrane and into the cytoplasm of hepatocytes^1–4^. Members of the organic anion transporting polypeptide family (OATPs) have a key role in this step^5–8^. They are expressed at the basolateral membrane, facing the space of Disse and the portal blood plasma, from where they mediate the uptake of a multitude of endogenous and exogenous compounds (Fig. 1)^6,9,10^. The endogenous substrates include steroid metabolites, intermediates of heme biosynthesis, the end product of heme breakdown (bilirubin) and its conjugates, and unconjugated and conjugated bile acids^6^. The exogenous substrates include a multitude of drugs including antivirals, chemotherapeutic agents, antidiabetics, or drugs used for treatment of cardiovascular diseases^1,9–12^. The latter class includes statins, which are widely prescribed for lowering plasma cholesterol levels for the reduction of the risk of atherosclerosis^13^.

Among the hepatic OATPs, OATP1B1 (gene name *SLCO1B1*) and OATP1B3 (gene name *SLCO1B3*) have been found to be of high clinical relevance. Impaired function of OATP1B1 or OATP1B3 due to genetic variations or inhibition is generally associated with adverse clinical events^1,4,11^. For example, genetic variants of the *SLCO1B1* gene have been associated with a higher incidence of adverse events in statin therapy, which required dose adjustments in patients^1,11^. Furthermore, co-administration of statins with OATP1B inhibitors such as cyclosporine or rifampicin can lead to increased systemic concentration of the victim drug, which can cause side-effects that range in severity from mild myopathy to life-threatening rhabdomyolysis^14–16^. Due to the large number of recorded clinical cases of such adverse events associated with reduced OATP1B function, regulatory agencies including the FDA, EMA, and PMDA recommend testing of newly developed drugs for potential OATP-mediated drug-drug interactions^17–20^.

OATP members are presumed to operate as either electroneutral or possibly electrogenic exchangers, and while no definitive proof was obtained, intracellular bicarbonate has been suggested as an endogenous counter-ion^6,8,9^. Furthermore, the transport activity of most OATPs was shown to be stimulated by acidic extracellular pH^21–28^. This is physiologically relevant because the space of Disse is likely acidic, as was suggested based on studies in rat livers^29^. While the role of liver OATPs in endogenous substrate transport and drug disposition is well established, their mechanism has remained unclear. This has been in part associated with a lack of high-resolution structures, which has impeded a detailed mechanistic understanding of substrate recognition, transport, and small-molecule inhibition.

In this study we present high-resolution structures of wild-type human OATP1B1 and OATP1B3 in distinct functional states. The structure of OATP1B1 bound to the endogenous substrate estrone-3-sulfate (E1S) shows how the architectural features of the substrate-binding pocket facilitate the recognition of structurally diverse anionic compounds. The structure of OATP1B3 reveals a binding pocket for a bicarbonate ion, which may serve as a counter-ion in the transport cycle. Our results enable us to predict the interaction of statins with OATP1B1 and OATP1B3 and provide a mechanistic explanation why certain OATPs mediate pH-sensitive transport.

## Results

### Functional expression of OATPs and generation of synthetic antibody fragments

**Fig. 2:**
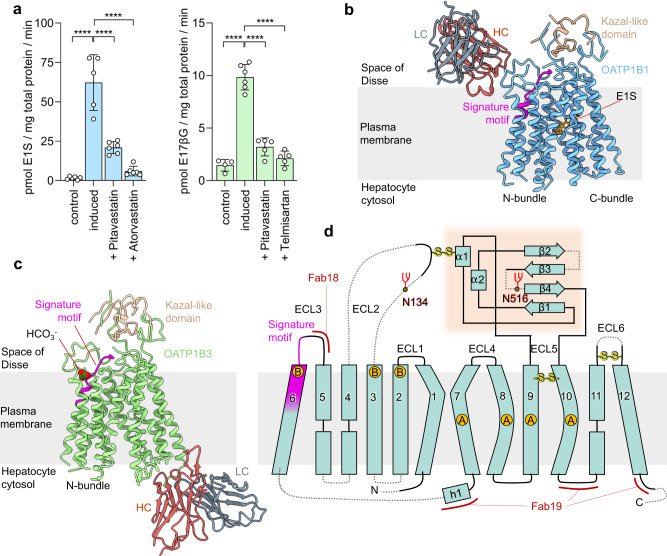
Functional analysis and structures of OATP1B1 and OATP1B3. **a** Cellular import of [^3^H]-E1S mediated by OATP1B1 (blue bars) or of [^3^H]-E17βG mediated by OATP1B3 (green bars). Non-induced (control) and tetracycline-induced stable cell lines were used for each protein. Total substrate concentrations were 1.0125 μM of E1S and 1.01 μM E17βG. Compounds were added at the following concentrations: 100 μM atorvastatin, 100 μM pitavastatin and 50 μM telmisartan. Bars represent means of *N* = 4, 5 or 6 biological replicates, error bars represent SDs. Statistical significance was determined using ordinary one-way ANOVA, multiple comparison test (Dunnett’s test at 95% CI) and differences are depicted as ****P ≤ 0.0001. **b** Structure of E1S-bound OATP1B1 in complex with Fab18. HC heavy chain, LC light chain of Fab18. **c** Structure of bicarbonate-bound OATP1B3 in complex with Fab19. HC, heavy chain; LC, light chain of Fab19. **d** OATP1B protein topology, with TM helices and extracellular loops (ECLs) numbered. Dotted lines indicate the flexible regions. The signature motif is colored magenta. The orange-shaded area denotes the Kazal-like domain. The two N-glycosylation sites (N134, N516) are indicated. The binding epitopes of Fab18 and Fab19 are depicted with red lines. Disulfide bonds in ordered regions are indicated by S-S for all regions except ECL5. Circles containing A indicate sites interacting with bound anionic substrate, and circles containing B indicate sites interacting with bound bicarbonate. PDB codes for OATP1B1 and OATP1B3 are 8PHW and 8PG0, respectively.

We expressed human OATP1B1 and OATP1B3 in stable, inducible HEK293 cells, confirmed that the proteins were localized at the plasma membrane, and tested their activity using cell-based transport assays (Fig. 2a). For OATP1B1, we observed uptake of radiolabeled estrone-3-sulfate (E1S), a conjugated sterol hormone and a physiological substrate of the transporter^9^. E1S contains pharmacophore features shared among OATP substrates: an anionic head group and a large hydrophobic moiety^30^. The activity of OATP1B3 was demonstrated by measuring uptake of [^3^H]-labeled estradiol-17-β-D-glucuronide (E17βG), a conjugated sterol that is a physiological substrate. To assess the interaction of the over-expressed transporters with exogenous compounds, we determined [^3^H]-E1S uptake by OATP1B1 in the presence of excess amounts of two statins, atorvastatin and pitavastatin. Under these conditions, [^3^H]-E1S uptake was abolished, demonstrating that these drugs compete with the observed transport activity of OATP1B1 (Fig. 2a). Similarly, we measured E17βG transport by OATP1B3 in the presence of excess amounts of pitavastatin and the blood pressure-lowering drug telmisartan. Both drugs abolished E17βG transport, demonstrating that they compete with the physiological substrate for OATP1B3 binding.

To achieve sufficiently high resolution for our structural studies, we isolated conformation-specific antigen-binding antibody fragments (Fabs) against OATP1B3 from a synthetic Fab library by phage display^31^. We identified two Fabs (Fab18 and Fab19) that form stable complexes with OATP1B3. Given that OATP1B1 and OATP1B3 share ~75% of sequence identity (Supplementary Fig. 1), we tested the Fabs for cross-reactivity and found that Fab18 was indeed able to form a stable complex with OATP1B1 (Supplementary Fig. 2). For structural studies, we reconstituted OATP1B1 and OATP1B3 into lipid nanodiscs, which mimic their native environment, and added the Fabs to serve as fiducials (Supplementary Fig. 3).

### Overall structures of OATP1B1 and OATP1B3

**Fig. 3:**
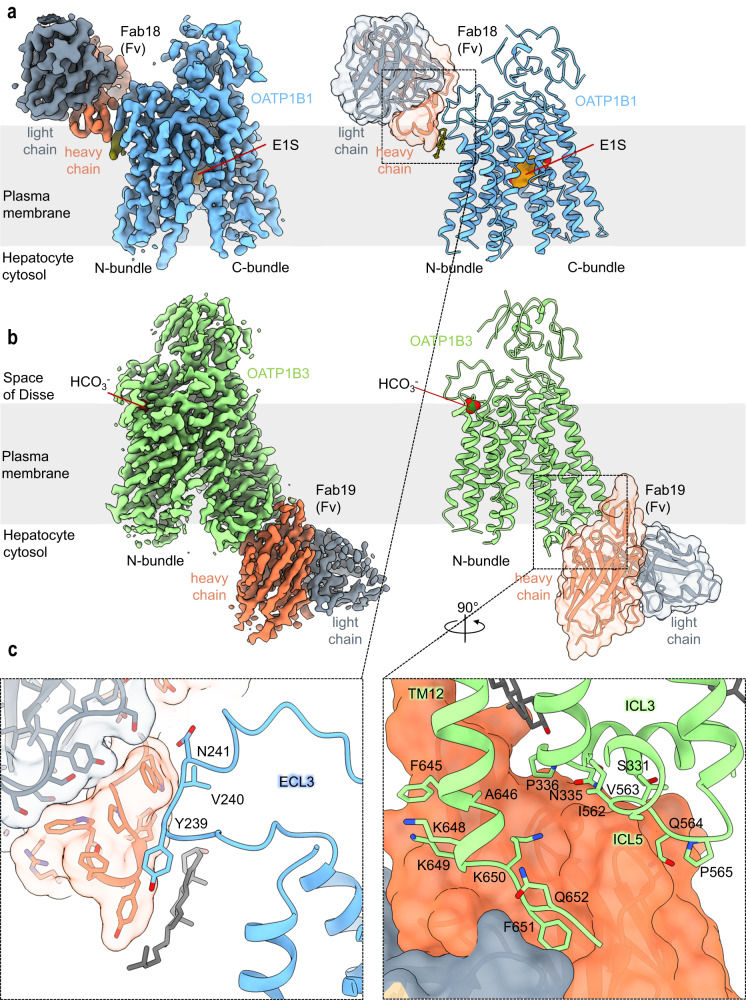
Fab-fragments facilitate structural study of OATP1B1 and OATP1B3. **a** EM density map (left) and ribbon representation (right) of the E1S-bound OATP1B1-Fab18 complex. Chains are colored separately and labeled. Fab fragment is also displayed as transparent surface. **b** EM density map (left) and ribbon representation (right) of bicarbonate-bound OATP1B3-Fab19 complex. The representation is shown as in (**a**). **c** Close-up views on binding epitopes of Fab18 (left) and Fab19 (right). The residues within 4.0 Å are displayed as sticks and labeled. An ordered cholesterol molecule is shown as grey sticks.

Using single-particle cryo-EM, we determined high resolution structures of OATP1B1 and OATP1B3. This was challenging for the OATP1B1-Fab18 complex because of the pronounced preferential orientation of the particles, which limited the resolution of the 3D reconstruction. We therefore collected additional data by tilting the specimen stage at angles of 20° and 30° and combined it with the data collected without tilting (Supplementary Fig. 4). This yielded an EM density map at 3.6 Å resolution, allowing for de novo model building (Fig. 2b). OATP1B1 features a major facilitator superfamily (MFS) fold, characterized by 12 transmembrane (TM) helices forming two pseudo-symmetrical bundles, termed N-bundle (TM1-TM6) and C-bundle (TM7-TM12) (Fig. 2b). Fab18 binds at the extracellular side of the transporter and interacts with a segment of ECL3 that is conserved in OATP1B3, explaining the cross-reactivity of this antibody fragment (Fig. 3a, c). The OATP1B1 structure revealed an inward-open conformation with a large, central cavity exposed to the hepatocyte cytosol. The cryo-EM analysis of OATP1B3 bound to Fab19 yielded an EM density map at 3.0 Å resolution (Supplementary Fig. 5). OATP1B3 also adopted an inward-open conformation (Fig. 2c), with Fab19 bound to the intracellular side of the C-bundle (Fig. 3b, c). However, there were structural differences associated with the distinct states the two transporters adopted (see below).

At the external side and exposed to the space of Disse, OATP1B1 and OATP1B3 both contain a large extracellular domain (ECD) with a mass of 25 kDa distributed over 4 extracellular loops (ECLs) and containing multiple disulfide bridges (Fig. 2d). The largest loop, ECL5, contains a Kazal-like domain^32^, which is cysteine-rich and has not been found in transporters other than in OATPs^8^. The Kazal-like domain is located above the C-bundle and was well-resolved in both structures. While similar domains were previously found in secreted serine protease inhibitors^32,33^, the function of this domain is currently unknown. Another unique feature of OATPs is the region located between the ECL3 and TM6, which contains the sequence motif D-x-RW-(I/V)-GAWW-x-G-(F/L)-L, where x denotes any amino acid residue. This motif is present in all OATPs and is therefore termed “OATP signature motif“^7,8^, and the two neighboring tryptophan residues were shown to be critical for activity^34,35^. The corresponding region was well-resolved in the EM density maps both of OATP1B1 and OATP1B3.

### Substrate-binding pocket

**Fig. 4:**
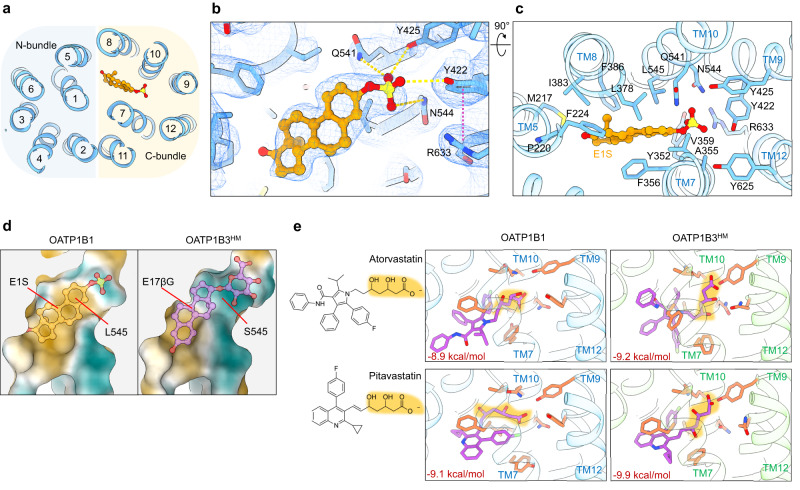
Drug binding pocket and statin docking. **a** Horizontal slice through a ribbon representation of E1S-bound OATP1B1, viewed from the external side, and with E1S shown as orange sticks. TM helices are numbered, and blue and yellow backgrounds depict the N- and C-terminus bundles, respectively. **b** EM density (blue mesh) of the OATP1B1 with fitted model shown from the membrane plane. Yellow lines indicate potential hydrogen bonds between the sulfate moiety and surrounding residues. Pink line indicates cation-π interaction **c** Residues within 5 Å of the bound E1S are shown as sticks and labeled. **d** Molecular lipophilicity potential. Surface representation of the substrate-binding pocket of E1S-bound OATP1B1 and the homology model of OATP1B3 with E17βG docked. Green and yellow indicate more hydrophilic and more hydrophobic areas, respectively. **e** Close-up views of the representative statin compounds docked in OATP1B1 (blue) and the homology model of OATP1B3 (green) structures using AutoDock Vina^37, 38^. The fits with the best overall docking scores (values in red) are displayed. Lactone moieties are shaded in orange. Residues for which flexibility of the side chains was allowed during docking are displayed as sticks.

**Fig. 5:**
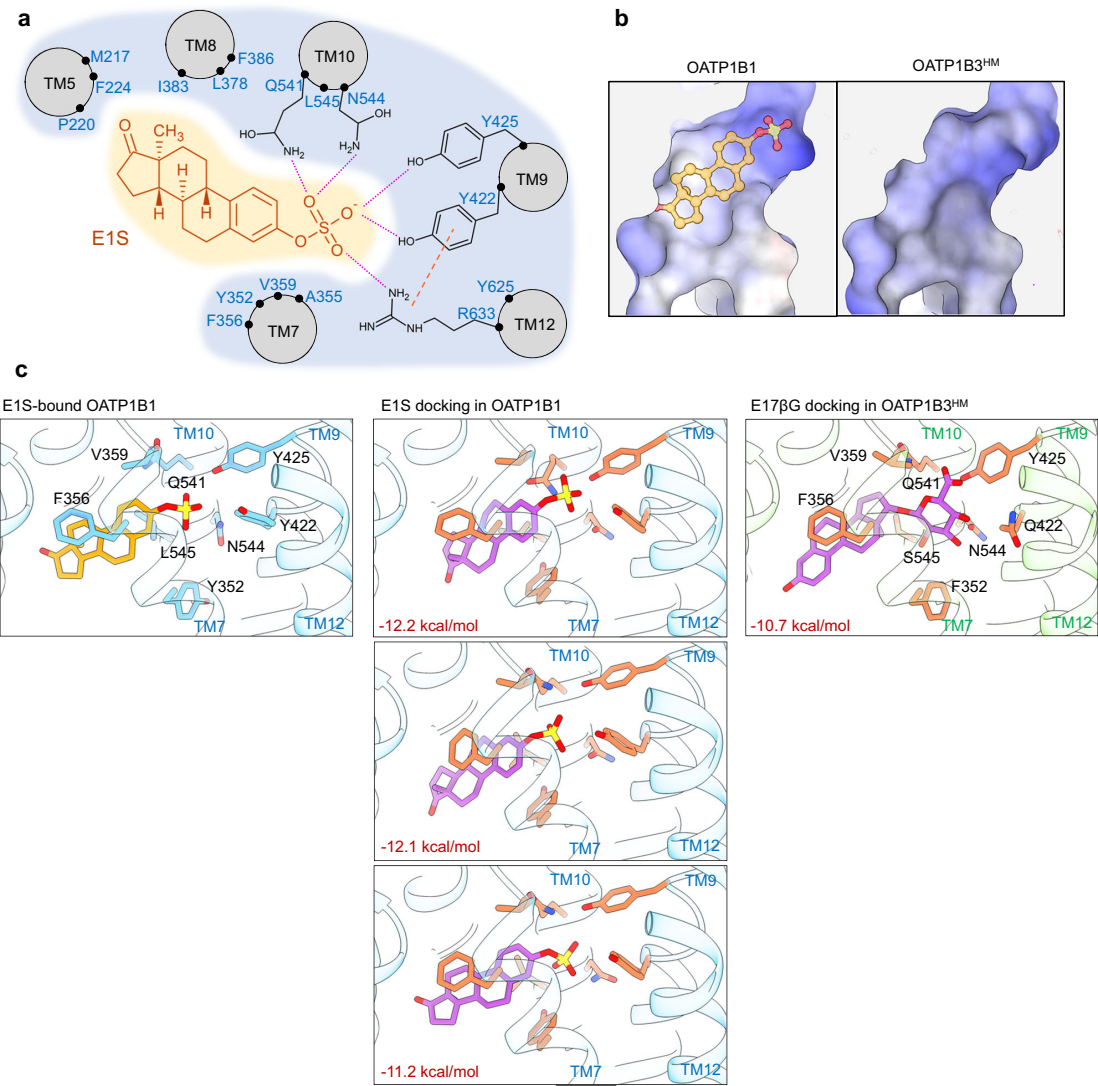
Characterization of the binding pocket of OATP1B1 and OATP1B3. **a** Road-kill plot presenting the interaction between bound E1S and the residues of OATP1B1. Hydrogen bonds are depicted as pink dotted lines. Cation-π interaction is shown as orange line. TM helices and interacting residues are labeled. **b** Electrostatic surface potential of the binding pocket of OATP1B1 and homology model of OATP1B3. Blue and red indicate positive and negative charges, respectively. White indicates neutral residues. **c** Molecular docking of E1S in OATP1B1 and E17βG in the homology model of OATP1B3 using AutoDock Vina^37, 38^. The fits with the best overall docking score are displayed (the best three fits are illustrated for E1S). E1S-bound structure (left) is displayed for comparison. Eight residues, for which flexibility was allowed in the molecular docking, are displayed at sticks. The residues of interest in both OATP1B1 and OATP1B3 and labeled.

The cryo-EM sample of OATP1B1 contained E1S, and we observed a density consistent with a single molecule of E1S bound in a funnel-shaped cavity located approximately half-way across the membrane and in the C-bundle of the transporter (Fig. 4a, b). The cavity is formed by residues of TM7, TM8, TM9 and TM10, and the E1S molecule is oriented with the negatively charged sulfate group at the apex of the cavity, while the sterol scaffold points towards the center of the transporter (Fig. 4a). The side chains of Tyr422, Tyr425, Gln541, and Asn544 form hydrogen bonds with the sulfate group of E1S. Among these, Tyr422 forms a cation-π interaction with the guanidinium group of Arg633, which can indirectly stabilize the negatively charged sulfate group of E1S (Fig. 4b). The remainder of the binding pocket of OATP1B1 is lined with aromatic and hydrophobic residues that contribute to Van der Waals interactions with the sterol scaffold of E1S (Fig. 4c, d). Among these, Leu545 has previously been reported to modulate substrate specificity in human OATP1B1^36^, which is in line with our structural observation. The shape and size of the binding pocket rationalize how OATP1B1 can recognize structurally distinct compounds that contain a negatively charged head group and a hydrophobic or aromatic body (Fig. 5a).

Given that our OATP1B3 structure did not contain bound substrate, we explored whether in silico molecular docking could rationalize substrate binding to OATP1B3. As a control, we used the program AutoDock Vina to perform molecular docking of E1S into the experimental structure of OATP1B1^37,38^. This resulted in two distinct binding poses, one of which was very similar to the experimentally determined structure, whereas in the other, the sterol moiety was rotated by 180° (Fig. 5c). This result provided confidence in the docking approach. Direct docking into the experimental structure of OATP1B3 was not possible: While there is a cavity in the structure, its size was too small for docking, which is a consequence of the transporter likely having been trapped and visualized in a state that is unable to bind substrates (see below). For molecular docking, we therefore generated a homology model of OATP1B3 based on our experimental OATP1B1 structure^39^. This revealed differences in the chemical nature of the cavity surface between the two transporters (Figs. 4d, 5b), as the region near the cavity apex contained more hydrophilic residues in OATP1B3 than in OATP1B1. For example, Leu545 of OATP1B1 corresponds to Ser545 in OATP1B3. Docking of the physiological substrate E17βG into OATP1B3 placed the negatively charged glucuronide moiety at the apex of the cavity, where it is stabilized through hydrogen bond interactions that are not present in OATP1B1 (Figs. 4d, 5c). Our results can thus rationalize an overlap in the substrate spectrum of OATP1B1 and OATP1B3 while explaining differences in their substrate specificity (Figs. 1, 2, 4d, 5b).

We sought to explore how OATP1B1 and OATP1B3 recognize statins as substrates and conducted molecular docking using our structures. Docking of atorvastatin and pitavastatin yielded good fits, with the negatively charged carboxylic acid moieties located at the apex of the binding cavity, where they are stabilized through a network of hydrogen bonds (Fig. 4e). The hydrophobic moieties of the statins adopted distinct poses in the funnel of the cavity. The docking scores of atorvastatin and pitavastatin are comparable to those of E1S or E17βG, suggesting that our in silico prediction of statins binding to OATP1B1 and OATP1B3 are likely of high confidence.

### Bicarbonate pocket in OATP1B3 structure

**Fig. 6:**
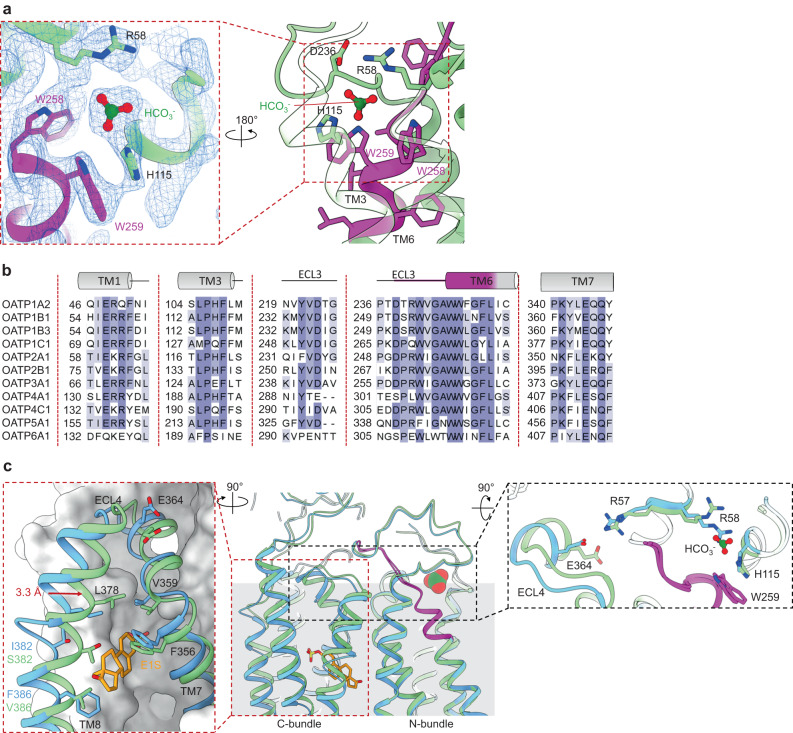
Bicarbonate binding pocket and allosteric changes. **a** Close-up views of the bicarbonate binding pocket in the OATP1B3 structure. Left panel depicts the EM density (blue mesh) assigned to the bicarbonate molecule. Residues in close contact with the HCO_3_^-^ anion are displayed as sticks and labeled. **b** Alignment of human OATP proteins. The sections correspond to the views presented in panel a. Secondary structural elements are depicted and labelled above the sequence. **c** Ribbon representation of E1S-bound OATP1B1 and bicarbonate-bound OATP1B3 structures superimposed on the N-bundle. (middle) View parallel to the membrane. Dotted frames indicate the zoomed-in regions. (left) Close-up view of the binding pocket with TM7 and TM8 displayed as ribbons. Red arrow indicates the shift in TM8. (right) Close-up view of the interface between the N- and C-terminal bundles showing a shift in ECL4.

The EM map of OATP1B3 revealed a well-resolved density feature located in the N-bundle of the transporter and at the level of the extracellular membrane boundary. Considering its flat, triangular shape and previous studies suggesting a potential role of bicarbonate as a co-substrate in OATP-mediated drug uptake^22^, we assigned this density feature to a HCO_3_^-^ molecule (Fig. 6a). The molecule is bound in a pocket lined by two conserved tryptophan residues of the OATP signature motif (Trp258 and Trp259) and interacts with the side chains of Arg58 and His115 (Fig. 6a, b). The latter is of particular importance because a mutation of the equivalent histidine in rat OATP1A1 (His130) to a glutamine was shown to abolish pH-sensitivity of transport^22^. A sequence alignment of the nine characterized and two orphan human OATPs (Fig. 6b) highlights the conservation of the residues forming the proposed HCO_3_^-^ binding pocket and reveals that His115 of OATP1B3 is present in all OATPs that mediate pH-dependent transport but absent in OATPs that are pH-independent (Supplementary Fig. 6). Based on the alignment, we predict that of the two orphan OATP proteins (bottom rows of the alignment in (Fig. 6b), OATP5A1 likely mediates pH-dependent transport, whereas OATP6A1 is expected to be pH-independent.

Given that the structures of OATP1B1 and OATP1B3 both revealed inward-facing conformations, we analyzed the EM density map of OATP1B1 for bound HCO_3_^-^. However, there was no density that suggests bound bicarbonate, and the side chains involved in HCO_3_^-^ binding in OATP1B3 adopt distinct conformations in OATP1B1. We then superimposed the N-bundle of OATP1B1 with that of OATP1B3, which yielded very good structural agreement (rmsd = 1.055 Å for C_α_ atoms in N-bundle). However, we observed structural differences at the interface of the N-bundle and the C-bundle as well as in TM7, TM8, and their connecting loop ECL4 (Fig. 6c). The bundle interface contains a salt bridge between the residues Arg57 and Glu364 (Fig. 6c), both of which are strictly conserved in OATP transporters (Fig. 6b). We found that TM7 and TM8 of OATP1B3 are shifted by ~3.3 Å (Fig. 6c) relative to the position of the corresponding helices in OATP1B1. Because these helices contain residues that line the E1S-binding cavity of OATP1B1, the observed changes have functional consequences. While the cavity in the OATP1B1 structure can bind E1S, that in OATP1B3 is too narrow to allow substrate binding. This suggests that allosteric changes, transmitted between the two TM bundles, might prevent OATPs from simultaneously binding a substrate in the C-bundle and a bicarbonate molecule in the N-bundle. Our OATP1B1 and OATP1B3 structures may thus have captured functionally distinct states that appear to be mutually exclusive, suggesting a mechanistic basis for understanding coupled transport.

## Discussion

Transport proteins of the major facilitator family, to which OATPs belong, are thought to operate by an alternating access mechanism^40^. In many cases, the architectural features include a central substrate-binding pocket at the shared interface of the N-bundle and the C-bundle. OATPs differ by featuring a binding pocket that is comprised exclusively in the C-bundle. While this is unusual, a similar feature has previously been observed for another vertebrate MFS transporter, the sodium-dependent lysophosphatidylcholine (LPC) transporter MFSD2A^41^. However, the remote site of bicarbonate binding and the apparent allosteric coupling with the drug-binding pocket in OATP1B3 have not been observed in MFS transporters to date. The key findings in our OATP1B1 structure are the location, shape and chemical nature of the drug binding pocket.

Given that one of the main functions of OATP1B1 and OATP1B3 is drug uptake, our OATP1B1 structure captured a state prior to cytoplasmic substrate release. We propose that the transport can be thought to involve at least three states. State I, represented by our E1S-bound OATP1B1 structure, features an inward-facing conformation and contains a substrate-filled, polyspecific binding pocket, allowing substrate release into the cytosol. State II, represented by our bicarbonate-bound OATP1B3 structure, features a narrowed substrate-binding pocket, a consequence of TM7 and TM8 adopting a more closed conformation (Fig. 6c). These conformational changes appear to be allosterically linked to the binding of bicarbonate near the OATP signature motif. In this conformation, substrate cannot re-bind to the central pocket. State II is likely followed by a switching to an outward-open conformation (proposed State III). This is essential to recruit another substrate from the environment, consistent with a rocker-switch-type alternating access model^40^. The release of bicarbonate, binding of a substrate molecule, and switching to an inward-open conformation would complete the cycle.

**Fig. 7:**
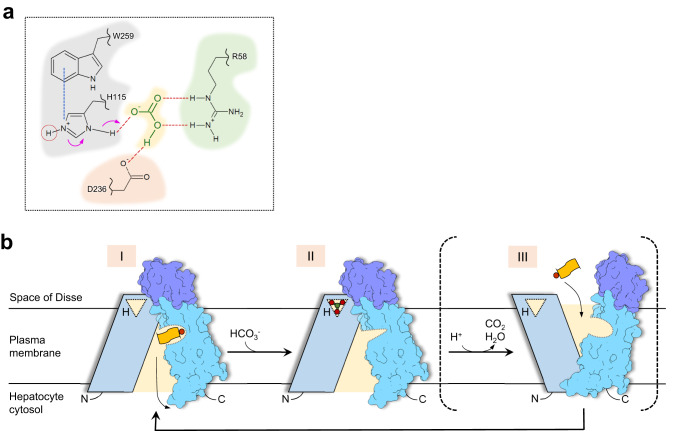
Protonation-dependent transport mechanism of organic anion substrates mediated by OATP. **a** Residues within the bicarbonate binding site are shown. Blue line indicates the cation-π interaction. Red lines show hydrogen bonds. Red circle indicates the hydrogen atom responsible for pH-sensitivity. **b** Proposed structure-based mechanism of transport of organic anion substrates mediated by pH-sensitive OATP proteins. The N- and C-terminal halves of the transporter are colored differently. The anionic moiety of the drug substrate (orange rectangle) is colored red. H indicates a key histidine residue responsible for mediating the protonation. States I and II represent structures from this study.

While the role of the primary substrate in this cycle is reasonably clear, that of bicarbonate is not. In the literature, OATP-mediated transport has been described both as electroneutral or possibly electrogenic. Electroneutrality would require a negatively charged ion to be counter-transported. For drug uptake, this would involve the extrusion of an ion extrusion, and HCO_3_^−^ might act as this counter-ion. If the transport is electrogenic, there might still be a role for bicarbonate. However, rather than a transport substrate, HCO_3_^-^ might act as an allosteric modulator of the transporter (Fig. 7). Future studies will be needed to clarify which scenario is the correct, or at least predominant one, in OATP1B1 and OATP1B3. It is important to mention that both scenarios are compatible with the experimentally observed trans-stimulation of OATP-mediated transport^5,42^.

The presence of a bicarbonate binding pocket near the OATP signature motif offers a simple explanation of why transport is stimulated by acidic external pH if the respective OATP protein contains a histidine residue. Bound bicarbonate forms several hydrogen bonds with the surrounding residues Arg58, His115 and Asp236 (Fig. 7a). Although the standard pKa value of histidine is 6.0^43^, the protonated form of His115 might be stabilized by a cation-π interaction with the adjacent tryptophan of the OATP signature motif (Trp259), which might cause an increase of the pKa of His115. Consequently, His115 is optimally positioned to accept a proton from the external side of the membrane and provide it to the bound bicarbonate. This would facilitate the conversion of HCO_3_^-^ into the less stable H_2_CO_3_, which likely dissociates into CO_2_ and H_2_O. We speculate that release of these degradation products could trigger the conversion of the transporter to the putative outward-open conformation (state III, Fig. 7b).

In conclusion, our results define the architecture of human OATP proteins and reveal structures of distinct states, allowing us to propose a mechanism of the transport cycle. The structures provide a framework for understanding and studying endogenous substrate and drug specificities of OATP1B1 and OATP1B3. Our mechanistic conclusions likely extend to other members of the OATP family such as the ubiquitously expressed OATP1A2 and OATP2B1. The obtained insight might provide opportunities for future drug design campaigns and for the prediction of drug–drug interactions, which might facilitate the re-evaluation and optimization of recommended drug dosage. Given the wide range of drugs transported by OATPs, our results will help rationalize drug disposition, specifically the different kinetics of drug uptake into the liver. An alternative application could be the design of OATP inhibitors to prevent toxin molecules like α-amanitin or microcystin to enter and damage liver cells, amounting to a small-molecule-based therapeutic approach in OATP-mediated toxic liver injury.

## Methods

### Construct design and protein expression

Human OATP1B1 (Uniprot: Q9Y6L6) and OATP1B3 (Uniprot: Q9NPD5) genes were codon-optimized for expression in human cells and purchased from GenScript. The synthetic genes were independently cloned in a pcDNA5 vector with a cleavable C-terminal YFP-1D4 tag. For protein expression, tetracycline-inducible stable cell lines (Flp-In™ T-REx™ 293 Cell Line, Thermo Fisher Scientific) were generated for each construct. Cells were grown and maintained in Dulbecco’s Modified Eagle Medium (DMEM, Gibco) supplemented with 10% fetal bovine serum (FBS, Thermo Fisher Scientific), 100 μg/mL streptomycin, 100 units/mL penicillin (Thermo Fisher Scientific) at 37 °C with 5% CO_2_ under humidified conditions.

Before protein expression, the media was aspirated, the cells were washed once with phosphate-buffered saline (PBS) buffer, and expression medium consisting of fresh phenol red dye-free DMEM (Gibco) supplemented with 2% FBS, 100 μg/mL streptomycin, 100 units/mL penicillin, 1x GlutMAX (Thermo Fisher Scientific), and 1x Sodium Pyruvate (Thermo Fisher Scientific), was added. Protein expression was induced with 1 μg/mL tetracycline (Sigma) at 37 °C. Following 72 h of expression, cells were harvested, washed with PBS, flash-frozen in liquid nitrogen, and stored at −80 °C for a maximum of 6 months.

### Cell-based transport experiments

Stable cell lines expressing OATP1B1 or OATP1B3 were independently seeded onto a 24-well plate (300,000 cells/well) previously coated with poly-D-lysine. Cells were grown for 18-24 h until they were 60–80% confluent. Before induction with 1 μg/mL tetracycline, the cells were washed with 1 mL of PBS. Afterwards, 0.75 mL/well of expression medium were added. For negative control, tetracycline was omitted. Following 24 h of expression, media was removed, and the cells were washed three times with 500 μL of pre-warmed Uptake Buffer (UB; 136 mM NaCl, 1.8 mM CaCl_2_, 1.1 mM KH_2_PO_4_, 0.8 mM MgSO_4_, 5.3 mM KCl, 11 mM D-glucose, and 20 mM HEPES, pH adjusted with 3 M Tris to pH 7.4). Transport was initiated by the addition into each well of 250 μL of UB containing 1.0125 μM of non-radiolabelled (Sigma) and radiolabelled (40 Ci/mmol, PerkinElmer) estrone-3-sulfate (E1S) for OATP1B1, and 1.01 μM non-radiolabelled (Sigma) and radiolabelled (46.3 Ci/mmol) estradiol-17-β-D-glucuronide (E17βG) for OATP1B3, at a molar ratio of 1:80 or 1:100 hot to cold substrate, respectively. For the competition assays, statins (100 μM atorvastatin or pitavastatin for OATP1B1 and 100 μM pitavastatin or 50 μM telmisartan for OATP1B3) were added to the UB. The uptake assay was stopped after 30 sec by aspirating the UB and immediately washing the cells three times with 500 μL ice-cold UB supplemented with 20 μM non-radiolabelled substrate. Cells were solubilized by adding 500 μL buffer composed of 2% Triton-X-100 and 1 M NaCl for 1 h at 37 °C. Total protein concentration per condition was measured in triplicates for OATP1B1, and duplicates for OATP1B3 using the BCA assay according to the manufacturer’s manual. For OATP1B1 350 μL of lysed cells, and for OATP1B3 450 μL were transferred to a 4 mL Scintillation Vial Ultima Gold (PerkinElmer). Intracellular radioactivity was measured by liquid scintillation counting after 24 h incubation. Data points compromised by technical errors were omitted from the analysis, resulting in an uneven number of replicates for different conditions. Data were processed and analyzed using ordinary one-way ANOVA, multiple comparison test (Dunnett’s test at 95% CI) in GraphPad Prism (version 9.0).

### OATP1B1 and OATP1B3 protein purification

Cell pellets were thawed at room temperature, broken by 25 strokes in a Dounce homogenizer in lysis buffer containing 25 mM HEPES pH 7.4, 150 mM NaCl, 20% (v/v) glycerol, 1 mM PMSF (phenylmethylsulfonyl fluoride), 2 μg/mL DNaseI (Roche), and protease inhibitor cocktail (Sigma). All subsequent steps were performed at 4 °C. Cell lysate was solubilized with 1% DDM (n-dodecyl-β-d-maltopyranoside, Anatrace), 0.2% (w/v) CHS (cholesteryl hemisuccinate, Anatrace) for 60–90 min. The solubilized lysate was ultracentrifuged at 140,000 × *g* in a Type 45-Ti rotor (Beckman) and the supernatant was incubated with pre-equilibrated Sepharose-coupled Rho-ID4 antibody (University of British Columbia) for 2–3 h. The resin was washed four times with ten column volumes (CV) of purification buffer containing 25 mM HEPES, pH 7.4, 150 mM NaCl, 20% glycerol, 0.025% DDM, and 0.005% CHS, followed by four washes with ten CV of wash buffer containing 25 mM HEPES, pH 7.4, 150 mM NaCl, 0.025% DDM, 0.005% CHS or supplemented with 10% glycerol for OATP1B1. After this step, OATP1B1 was eluted from the resin with 3 CV of wash buffer supplemented with 0.5 mg/mL 1D4 peptide following overnight incubation. OATP1B3 was eluted by incubating the resin with 3 CV of wash buffer containing 3C protease at a 1:50 wt/wt ratio for 1 h at 4 °C.

### Nanodisc reconstitution

Pre-mixed lipids (Brain Polar Lipid Extract, Avanti) with cholesterol (Avanti) were solubilized with detergent and added to purified OATP1B1 or OATP1B3 and incubated for 10 min at room temperature. Purified membrane scaffold protein MSP1D1 was added to the mixture and incubated for an additional 20 min at room temperature. Bio-Beads SM-2 were pre-washed with HBS (25 mM HEPES, pH 7.4, 150 mM NaCl) and added before overnight incubation. Nanodisc-reconstituted OATP1B3 was pre-mixed with Fab19 at a molar ratio of 1:1 and the OATP1B3-Fab19 sample was further purified by size-exclusion chromatography in HBS prior to sample freezing. For OATP1B1, the eluted mixture was incubated for 5 h with pre-equilibrated Sepharose-coupled Rho-ID4 antibody and washed four times with 3 CV of HBS to remove empty nanodiscs. The purification tag was removed by incubating for 2 h with 3C protease. Eluted mixture was premixed with 1:1.75 molar excess Fab18 prior to size-exclusion chromatography step (Superdex 200 Increase 10/300 column, GE Healthcare) in HBS buffer to remove excess Fab.

### OATP1B3 biotinylation

An OATP1B3 expression construct containing an Avi-tag sequence (GLNDIFEAQKIEWHE) was generated using PCR-restriction cloning (forward primer 5′-TCG ACG GGT TGA ATG ATA TTT TCG AAG CAC AGA AAA TTG AAT GGC ATG AGG − 3′ and reverse primer 5′-CGG GTT GAA TGA TAT TTT CGA AGC ACA GAA AAT TGA ATG GCA TGA GGT CGA − 3′). Protein expression and purification were performed as described above with the following changes. Cell pellets were lysed and subsequently solubilized with 1% LMNG (lauryl maltose meopentyl glycol, Anatrace), 0.2% CHS (w/v). Following elution with 3C protease to remove the purification tag, the sample was enzymatically biotinylated using in-house purified BirA, as described previously^44^. Briefly, the OATP1B3 Avi-tag construct was incubated overnight at a 1:1 molar ratio with in-house purified BirA to protein in buffer supplemented with 0.02% LMNG, 0.004% CHS, 250 μM biotin, 10 mM ATP, 50 mM Bicine, pH 8.3, and 10 mM Mg Acetate. Biotinylation efficiency of the sample was verified by a pull-down assay on streptavidin-coated (SA) magnetic particles (Promega). Biotinylated OATP1B3 was purified by size-exclusion chromatography in HBS supplemented with 0.02% LMNG and 0.004% CHS.

### Phage display selection

Biotinylated OATP1B3 was used for phage display selection. Phage display selection was performed at 4 °C according to published protocols^45^. The selection buffer consisted of 25 mM HEPES, pH 7.4, 150 mM NaCl, 0.02% LMNG, 0.004% CHS, and 0.5% BSA. In the first round, 250 nM of target was immobilized on 250 µL SA magnetic beads. Then, 100 μL of a phage library E were added to the target bound to the SA beads and incubated for 30 min^46^. The resuspended beads containing bound phages were washed extensively and then used to infect log phase *E.coli* XL1-Blue cells. Phages were amplified overnight in 2xYT media with 50 µg/mL ampicillin and 10^9^ p.f.u./mL of M13-KO7 helper phage. To obtain binders of high affinity and specificity, four additional rounds of selection were performed with decreasing the target concentration in each round with the final concentration of the target being 25 nM in the 5^th^ round of selection. In each round, the amplified pool of phages of the preceding round was used as the input. From the second round onwards, the bound phages were eluted using 100 mM glycine, pH 2.7. This elution technique often results in the elution of non-specific and Streptavidin binders. To eliminate them, the precipitated phage pool from the second round onwards were negatively selected against 100 µL of SA magnetic beads before adding them to the target. The pre-cleared phage pool was then used as input for the selection.

### Single-point phage ELISA

The ELISA experiments were performed at 4 °C in 96-well plates coated with 50 µL of 2 µg/mL neutravidin in Na_2_CO_3_ buffer, pH 9.6 and subsequently blocked by 0.5% BSA in PBS. A single-point phage ELISA was used to rapidly screen the binding of the obtained Fab fragments displayed on phage. Colonies of *E.coli* XL1-Blue harboring phagemids from 5th round of selection were inoculated directly into 500 μL of 2xYT broth supplemented with 100 μg/mL ampicillin, and M13-KO7 helper phage. The cultures were grown overnight at 37 °C in a 96-deep-well block plate. The ELISA buffer was identical to that used in selection. The experimental wells in the ELISA plates were incubated with 50 nM OATP1B3 in ELISA buffer for 15 min. Only buffer was added to the control wells. Overnight culture supernatants containing Fab phage were diluted 10-fold in ELISA buffer. The diluted phage supernatants were then transferred to ELISA plates that were pre-incubated with biotinylated target and washed with ELISA buffer. The ELISA plates were incubated with the phage for another 15 min and then washed with ELISA buffer. The washed ELISA plates were incubated with a 1:1 mixture of mouse anti-M13 monoclonal antibody (GE HealthCare, 1:5000 dilution in ELISA buffer) and peroxidase conjugated goat anti-mouse IgG (Jackson Immunoresearch, 1:5000 dilution in ELISA buffer) for 30 min. The plates were washed again, developed with TMB substrate, then quenched with 1.0 M HCl, and the absorbance at 450 nm was determined. The background binding of the phage was monitored by the absorbance from the control wells.

### Sequencing, cloning, expression and purification of Fab fragments

From phage ELISA, clones (selected based on a high ratio of ELISA signal of target binding to background) were sequenced at the DNA Sequencing Facility at the University of Chicago. Twelve unique clones were obtained. These were sub-cloned in pRH2.2, an IPTG inducible vector for expression of Fabs in *E. coli*. *E. coli* C43 (Pro^+^) cells were transformed with sequence-verified clones of Fab fragments in pRH2.2^47^. Fab fragments were grown in TB autoinduction media with 100 μg/mL ampicillin overnight at 30 °C. Harvested cells were kept frozen at −80 °C until use. Frozen pellets were re-suspended in PBS supplemented with 1 mM PMSF, and 1 μg/mL DNaseI. The suspension was lysed by ultrasonication. The cell lysate was incubated at 65 °C for 30 min followed by centrifugation. The supernatant was filtered through 0.22 µm filter and loaded onto a HiTrap Protein L 5-mL column pre-equilibrated with lysis buffer (20 mM HEPES buffer, pH 7.4, 500 mM NaCl). The column was washed with 10 column volumes of lysis buffer followed by elution of Fab fragments with elution buffer (100 mM acetic acid). Fractions containing protein were directly loaded onto a Resource S 1-mL column pre-equilibrated with buffer A (50 mM sodium acetate, pH 5.0) followed by washing with 10 column volumes wash with buffer A. Fab fragments were eluted with a linear gradient 0–50% of buffer B (50 mM sodium acetate, pH 5.0, 2.0 M NaCl). Affinity and ion-exchange chromatography were performed using an automated program on ÄKTA explorer system. Purified Fabs were dialyzed overnight against 20 mM HEPES, pH 7.4, 150 mM NaCl. The quality of purified Fab fragments was analyzed by SDS–PAGE.

### Multipoint protein ELISA for EC_50_ determination

Multipoint ELISA was performed at 4 °C to estimate the affinity of the Fabs to OATP1B3. 25 mM HEPES, pH 7.4, 150 mM NaCl, 0.02% LMNG, and 0.004% CHS supplemented with 0.5% BSA was used as the ELISA buffer. 50 nM of target immobilized on a neutravidin coated ELISA plate was incubated with 3-fold serial dilutions of the purified Fabs starting from 4 μM for 20 min. The plates were washed, and the bound target-Fab complexes were incubated with a secondary HRP-conjugated Pierce recombinant protein L (Thermo Fisher Scientific, 1:5000 dilution in ELISA buffer) for 30 min. The plates were again washed, developed with TMB substrate, quenched with 1.0 M HCl, and absorbance (A_450_) was determined. To determine the affinities, the data were fitted in a dose-response sigmoidal function in GraphPad Prism and EC_50_ values were calculated.

### Cryo-EM studies

Cryo-EM grids were prepared using a Vitrobot Mark IV (FEI) with an environmental chamber set at 95% humidity and 4 °C. Aliquots of 3.5 μL of purified OATP1B1 in complex with Fab18 in presence of 0.065 mM E1S or OATP1B3:Fab19 complex in presence of 0.05 mM atazanavir at a protein concentration of 0.22 mg/mL and 0.54 mg/mL respectively, were placed onto Quantifoil carbon grids (R1.2/1.3, 300 mesh, copper) previously glow-discharged for 45 s with 25 mA using Pelco easiGlow Glow Discharge Cleaning System. Grids were blotted for 3.5 s and flash-frozen in a mixture of liquid ethane and propane cooled by liquid nitrogen.

Grids were imaged with a Titan Krios (Thermo Fisher Scientific) electron microscope operated at 300 keV, equipped with a Gatan K3 Summit direct electron detector and Gatan Imaging Filter (GIF), with a slit width of 20 eV to remove inelastically scattered electrons. Movies were recorded semi-automatically with EPU2 software (Thermo Fisher Scientific) in super-resolution counting mode with a defocus range of –0.6 to –2.2 μm and a super-resolution pixel size of 0.255 Å/pixel. The final E1S-bound OATP1B1-Fab18 dataset consisted of 21,416 super-resolution movies collected during three separate Titan Krios sessions, including 20 degrees and a 30 degrees tilted datasets containing 1424 and 7041 movies, respectively. Tilted datasets were collected to overcome the problem of particle preferred orientation. Each movie was exposed for 1.5 s with an exposure time of 0.03 s per frame with electron exposure of 10.5 e-/pix/s, resulting in 50 frames per movie and a frame exposure rate of 1.2 e-/Å^2^. The final OATP1B3-Fab19 dataset was composed of 25,887 super-resolution movies collected during two cryo-EM sessions. Movie stacks had identical exposure time, dose, and frame number as the the E1S-bound OATP1B1-Fab18 dataset.

### Image processing

The analyses of data processing are presented in Supplementary Figs. 4 and 5. Briefly, the multiframe micrographs from all datasets were processed in Relion 4 starting with the motion correction (MotionCor2), the dose weighting and binning by a factor of 2 (resulting in a pixel size of 0.51 Å/px)^48,49^. Gctf was used to estimate the parameters of contrast transfer function (CTF)^50^ The micrographs were automatically sorted in Relion, and only those with an estimated resolution lower than 4 Å (or lower than 5 Å for datasets with tilt stage) were selected for further processing. First, particles were autopicked using Laplacian-of-Gaussian filtering and 2D classified in several rounds, followed by a generation of ab initio models of both OATP1B1-Fab18 and OATP1B3-Fab19 complexes. These models were used accordingly as template for the particles autopicking. Extracted particles were binned by a factor of 4 and used for several rounds of 2D and 3D classifications. From each dataset the 3D classes that most closely represented the expected structure of the complexes were selected.

For the OATP1B1-Fab18 dataset, the combined particles from three datasets (737,734 particles) were re-extracted (unbinned to 0.51 Å/px) and used for 3D classification. The class with the well-resolved TMD region was selected (198,430 particles) and used for another 3D classification. Then the class, in which ICLs were well-resolved, was selected, 3D refined and CTF refined. The particles (104,547) were Bayesian polished (first 20 frames), again CTF refined and 3D refined using mask that excluded the density of the nanodisc and the constant domain (Fc) of the Fab18. The final EM density map generated in Relion yielded 3.55 Å resolution. Next, the shiny particles from Relion were transferred to CryoSPARC4.1.^51^ The NU-refinement, followed by the Local CTF refinement and another NU-refinement yielded the final map at 3.67 Å resolution. The half-maps from the last 3D refinement were sharpened and post-processed with DeepEMhancer.^52^

For the OATP1B3-Fab19 complex dataset, the combined particles from two datasets (729,787 particles) were used for 3D classification. The class with the well-resolved TMD and ECD regions was selected (287,710 particles) and the particles were re-extracted (unbinned to 0.51 Å/px), followed by the 3D refinement. Then the particles were 3D classified without alignment and with masking out the nanodisc and constant domain (Fc) of the Fabs. Among the four classes, we did not observe more than a single conformation or any density indicating the presence of the atazanavir molecule. Therefore, the class that showed good connectivity of the signal and the best resolved TMD and ECD regions was selected for further processing. The 75,610 particles were 3D refined, Bayesian polished (first 15 frames), CTF refined, 3D refined and post-processed (B-factor sharpened) yielding the final EM density map at 2.97 Å resolution.

### Model building and refinement

Model building for both OATP1B1 and OATP1B3 was performed using Coot^53^. Both maps were of sufficient resolution to allow de novo building of the transmembrane regions and parts of the extracellular domains of the transporters. The variable domain of the Fab binders were modelled de novo in Coot and the chemical structures for E1S, HCO_3_^−^ and cholesterol were obtained from the monomer library available in Coot, using the codes FY5, BCT and CLR, respectively. The generated models were refined in Phenix using Real Space Refinement, and the quality was assessed by MolProbity. Further analyses of the EM density map and the model for each structure are presented in Supplementary Figs. 7 and 8. Q-scores were calculated with the MapQ plug-in in UCSF Chimera. The directional FSC plots were generated on the remote 3DFSC Processing Server^54,55^.

### Ligand docking

The chemical structures of the compounds (E1S, statins, estradiol 17β-D-glucuronide) were taken from the Zinc15 database (https://zinc15.docking.org/) or PubChem (https://pubchem.ncbi.nlm.nih.gov/). If necessary, the carboxyl group was modified (deprotonated) in JSME Molecular Editor (Zinc15) and the SMILES strings were copied. The SMILES were converted to SDF format in OpenBabel^56^. The ligand PDBQT files were generated from SDF files using the Meeko python package (mk_prepare_ligand.py, with default settings: add Gasteiger partial charges, merge non-polar hydrogen atoms, assign atom types). The protonation state of the ligands was checked before docking. For molecular docking with flexible side chains, we used the PDBQT ligand files.

Flexible docking was performed using Autodock Vina as described in the online tutorial (https://autodock-vina.readthedocs.io/en/latest/docking_flexible.html#). Briefly, docking was performed with either our current OATP1B1 model (the E1S was removed) or the OATP1B3 homology model based on our OATP1B3 and OATP1B1 structures built in SWISS-MODEL (https://swissmodel.expasy.org/). The models were prepared for the docking using Autodock Vina with prepare_receptor with the default settings; the missing hydrogen atoms (prepare_receptor, -A “hydrogens” flag) and Gasteiger charges were added to the models.

For each of the OATP1B transporters, a set of eight flexible residues from TM7, TM9 and TM10 involved in the substrate binding was selected, i.e., Phe356, Tyr352, Val359, Tyr422, Tyr425, Asn544, Leu545, Gln541 for OATP1B1, and Phe356, Phe352, Val359, Gln422, Tyr425, Asn544, Ser545, Gln541 for OATP1B3. The rigid and flexible parts of the transporters were calculated (prepare_flexreceptor.py, -s flag). The Vina forcefield was used for the analyses. The center and dimensions of the grid space were defined (in Angstrom) Grid center: X 120 Y 130 Z 110. Grid size: X 30 Y 30 Z 30. An exhaustiveness of 64 was used for a more extensive sampling of poses. Nine poses were generated for each system and classified based on their affinity scores (in kcal/mol). Autodock Vina wrote the PDBQT output files which were displayed in ChimeraX using the interactive ViewDockX tool.

### Figure preparation

Figures were prepared using the programs ChimeraX, PyMOL (The PyMOL Molecular Graphics System, DeLano Scientific), and GraphPad. Multiple sequence alignments were generated using Clustal Omega online software^57^. Chemical structures were prepared using ChemDraw (Revvity Signals Software).

### Reporting summary

Further information on research design is available in the Nature Portfolio Reporting Summary linked to this article.

### Supplementary information

Supplementary Information

Peer Review File

Reporting Summary

### Source data

Source Data

## Data Availability

Atomic coordinates of the E1S-bound OATP1B1-Fab18 and bicarbonate-bound OATP1B3-Fab19 models have been deposited in The Protein Data Bank (PDB) under accession numbers 8PHW, 8PG0. The three-dimensional cryo-EM density postprocessed, masked maps and half-masks have been deposited in the Electron Microscopy Data Bank (EMDB) under accession number EMD-17677 and EMD-17655. All other data are available from the corresponding author upon request. Source data are provided with this paper.
