# Supplementary material for: Structure of human drug transporters OATP1B1 and OATP1B3
Source: Nat Commun. 2023 Sep 18;14:5774. doi: 10.1038/s41467-023-41552-8 (PMC10507018; doi:10.1038/s41467-023-41552-8)
Supplement: Supplementary file 1 — Supplementary Information [file 41467_2023_41552_MOESM1_ESM.pdf]

SUPPLEMENTARY INFORMATION FOR:

Structure of human drug transporters OATP1B1 and OATP1B3

Anca-Denise Ciută[1,3], Kamil Nosol[1,3], Julia Kowal[1,3], Somnath Mukherjee[2,3], Ana S. Ramírez[1], Bruno Stieger[1], Anthony A. Kossiakoff[2], Kaspar P. Locher[1†]

[1] Institute of Molecular Biology and Biophysics, Department of Biology, ETH Zürich, Zürich, Switzerland
[2] Department of Biochemistry and Molecular Biology, The University of Chicago, Chicago, IL, USA
[3] Authors have contributed equally

[†] To whom correspondence should be addressed: locher@mol.biol.ethz.ch

This file contains:

Supplementary Figures 1-8
Supplementary Table 1

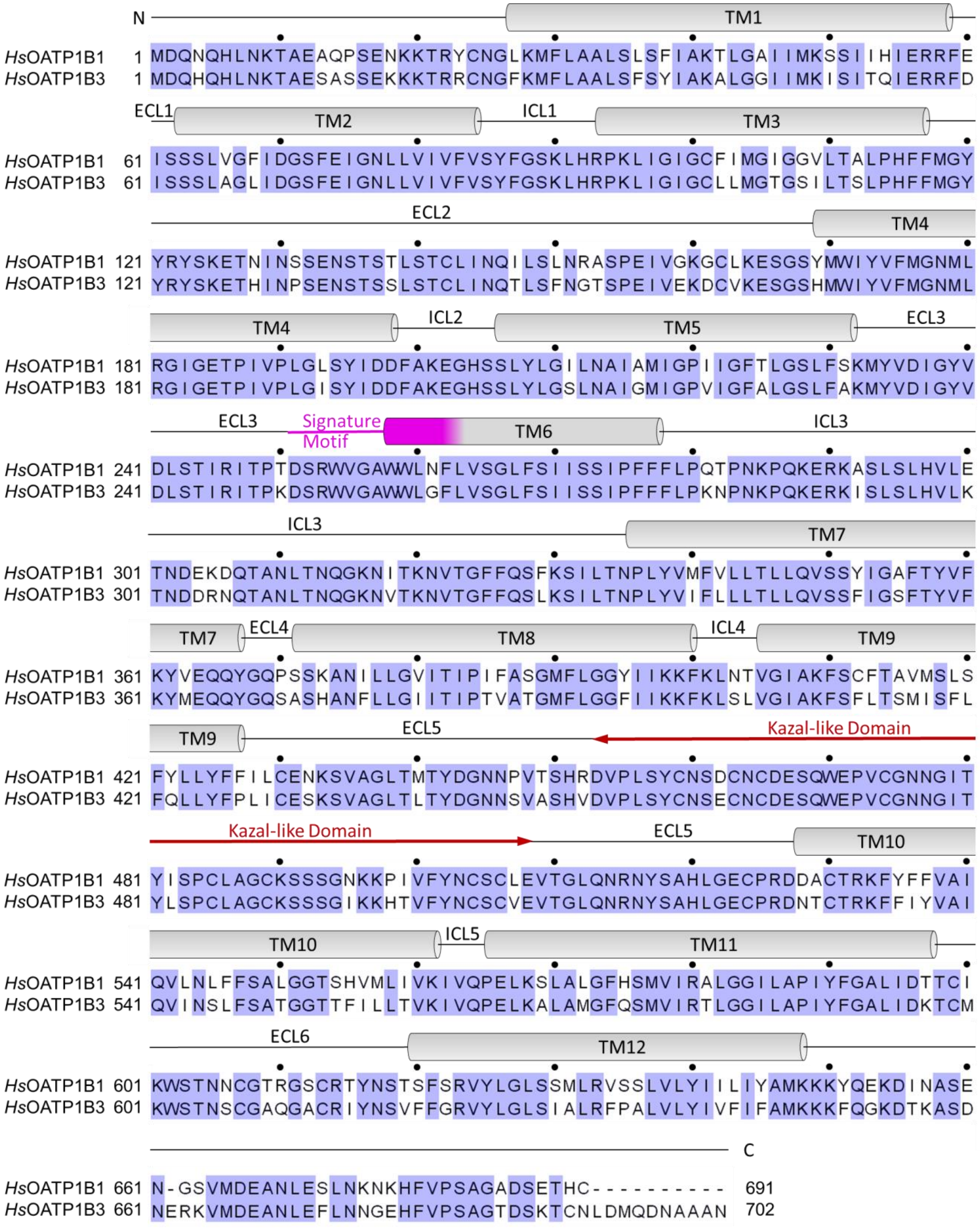

Supplementary Fig. 1. Sequence alignment of OATP1B1 and OATP1B3.
Alignment of human OATP1B1 (*Homo sapiens* OATP1B1, ID: Q9Y6L6) and human OATP1B3 (*Homo sapiens* OATP1B3, ID: Q9NPD5) sequences, generated in Clustal Omega[57]. The motifs and secondary structure elements are depicted. TM - transmembrane helix; ECL - extracellular loop; ICL - intracellular loop.

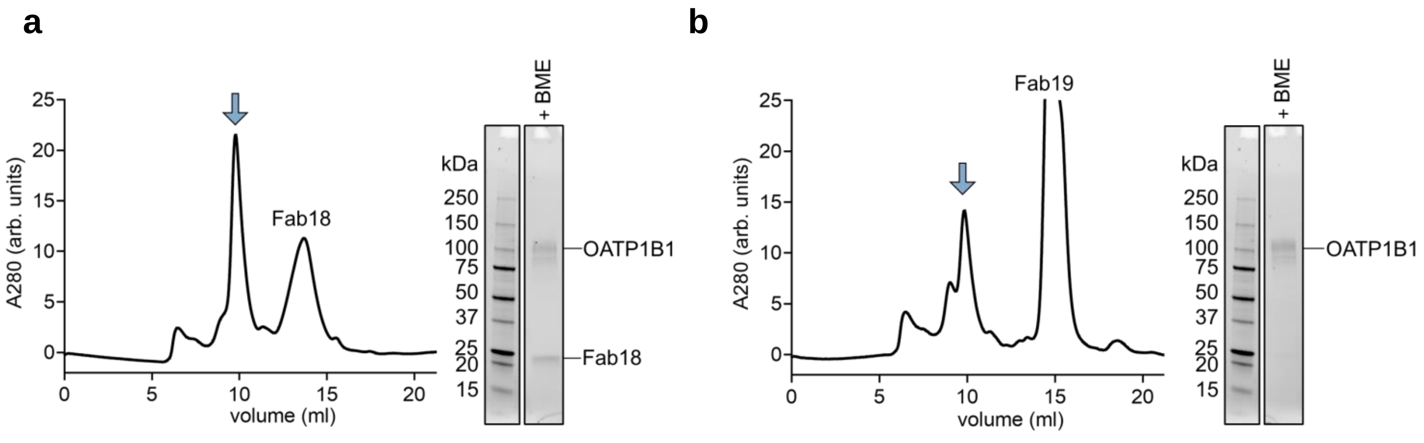

Supplementary Fig. 2. Testing cross-reactivity between OATP1B3 Fabs and antigen OATP1B1.
a Complex formation of detergent-solubilized OATP1B1 and Fab18. Blue arrow indicates the fraction used SDS-PAGE analysis in the presence of 2-mercaptoethanol. **b** Complex formation of detergent-solubilized OATP1B1 and Fab19. Blue arrow indicates the fraction used SDS-PAGE analysis in the presence of 2-mercaptoethanol. The SDS-PAGE image of the ladder and the sample originate from the same gel image.

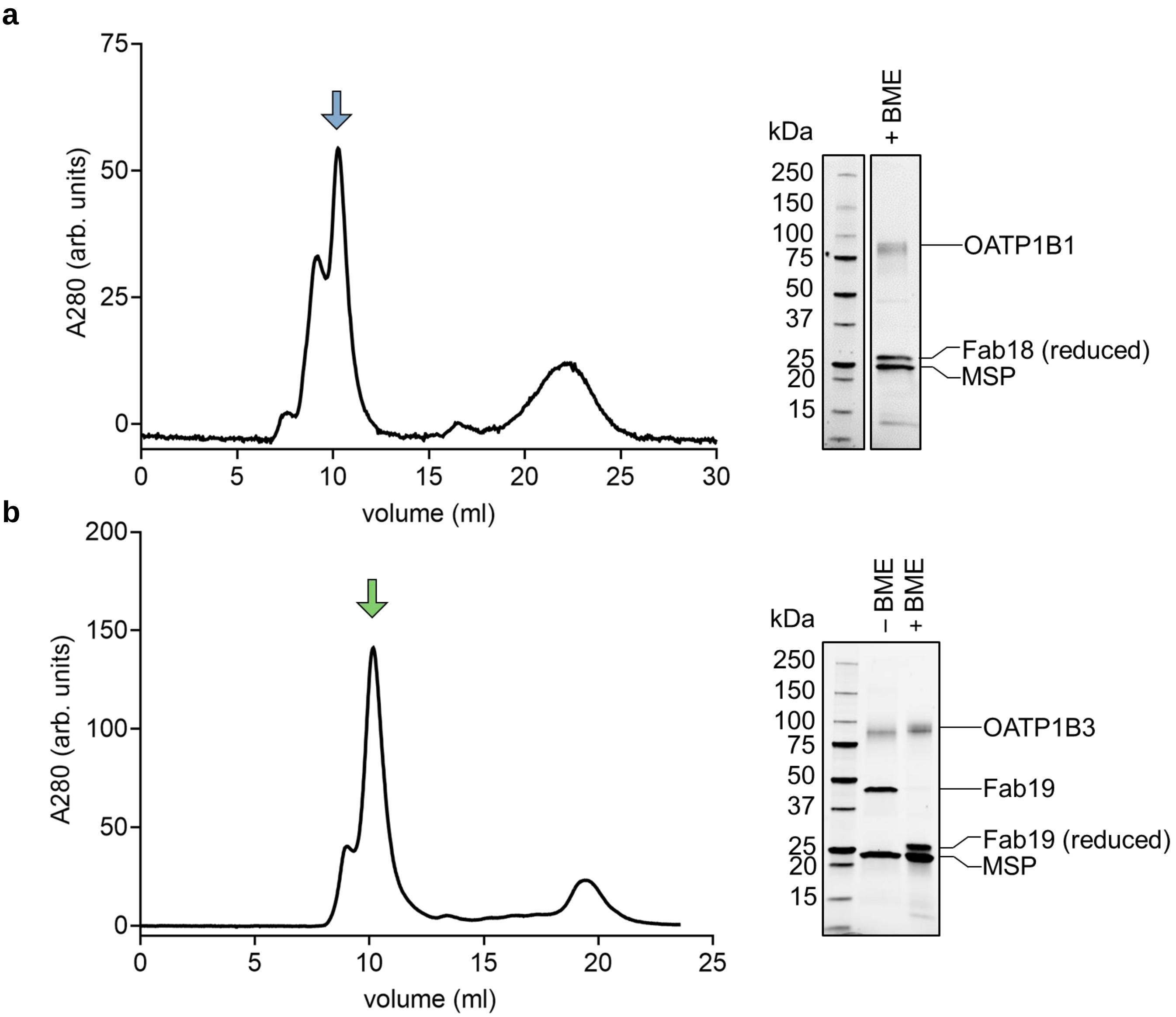

Supplementary Fig. 3. Sample preparation of OATP1B1-Fab18 and OATP1B3-Fab19 for cryo-EM analysis.
a Size exclusion chromatography (SEC) of OATP1B1-Fab18. Blue arrow indicates the fraction used for grid preparation. SDS-PAGE analysis of the pooled fraction in the presence of 2-mercaptoethanol (BME). The SDS-PAGE image of the ladder and the sample originate from the same gel image. **b** Size exclusion chromatography (SEC) of OATP1B3-Fab19. Green arrow indicates the fraction used for grid preparation. SDS-PAGE analysis of the pooled fraction in the absence and presence of 2-mercaptoethanol.

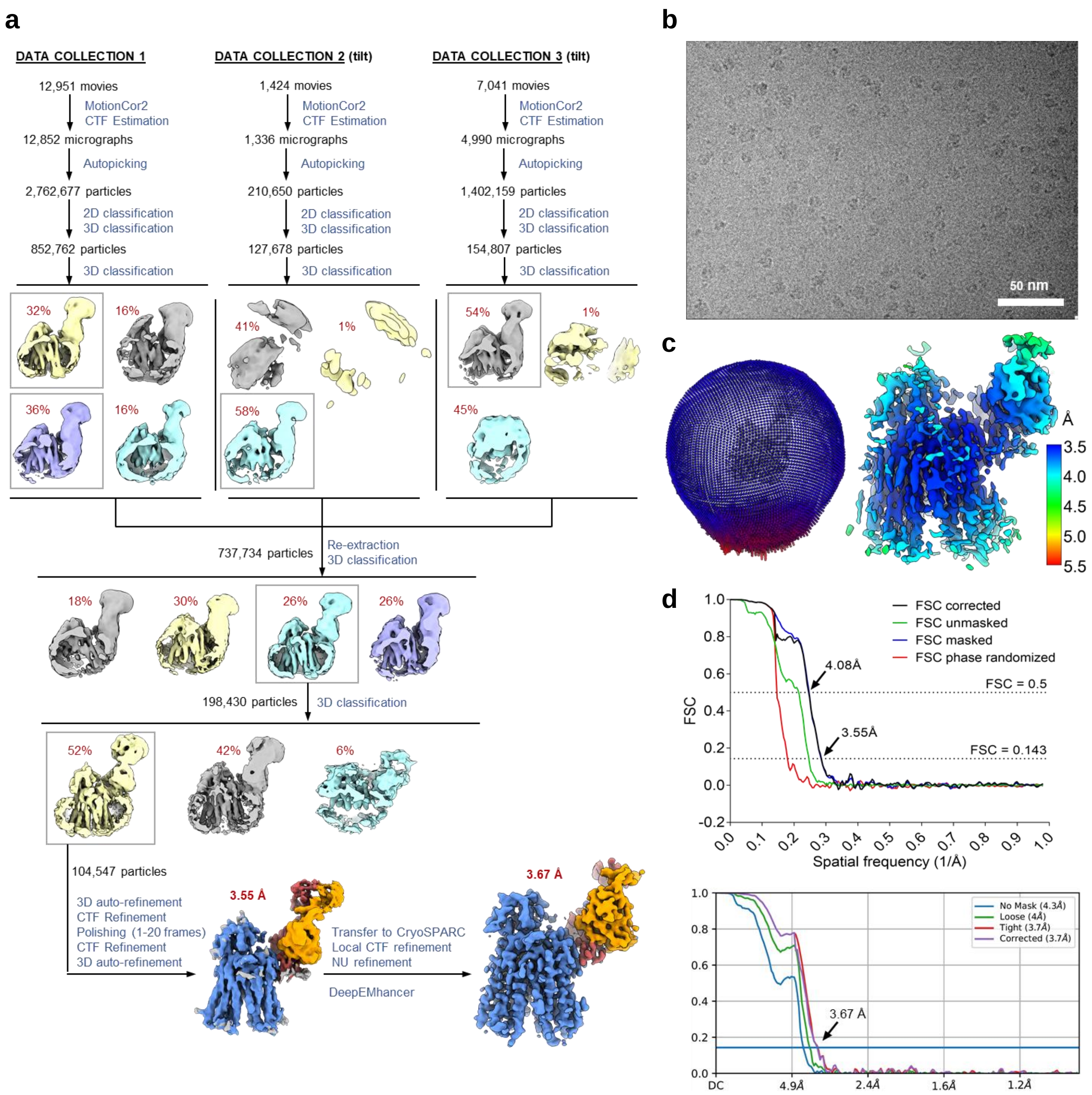

Supplementary Fig. 4. Cryo-EM analysis of E1S-bound OATP1B1 in a complex with Fab18.
a Flowchart presenting the pipeline of data processing in Relion 4. **b** Representative micrograph of imaged sample. **c** Angular distribution of the particles (*left*) and local resolution estimates of the final EM density map (*right*). **d** FSC curves of the final EM density maps calculated in Relion 4 (top) and cryoSPARC (bottom).

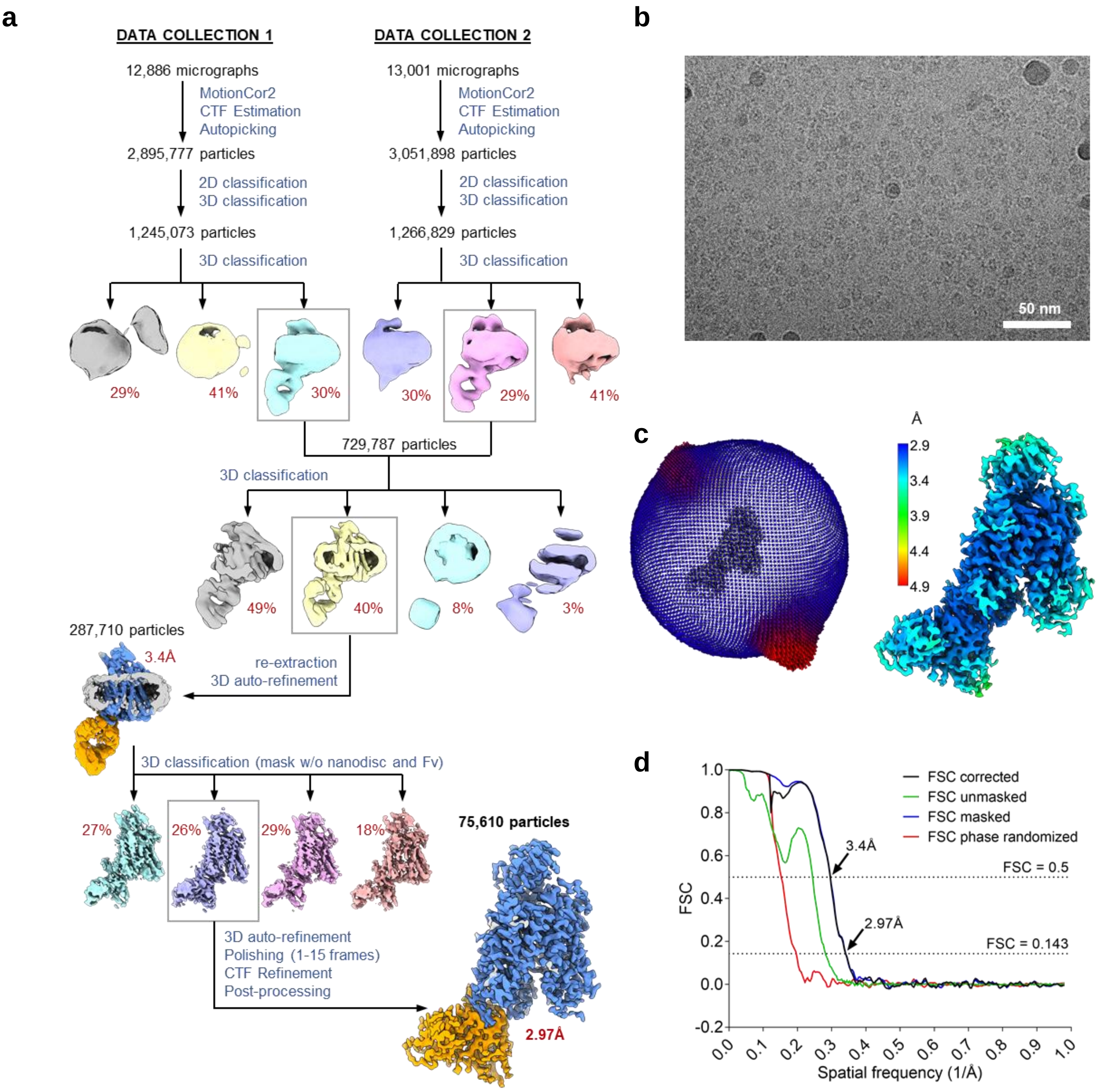

Supplementary Fig. 5. Cryo-EM analysis of bicarbonate-bound OATP1B3 in a complex with Fab19.
a Flowchart presenting the pipeline of data processing in Relion 4. **b** Representative micrograph of imaged sample. **c** Angular distribution of the particles (*left*) and local resolution estimates of the final EM density map (*right*). **d** FSC curves of the final EM density maps calculated in Relion 4.

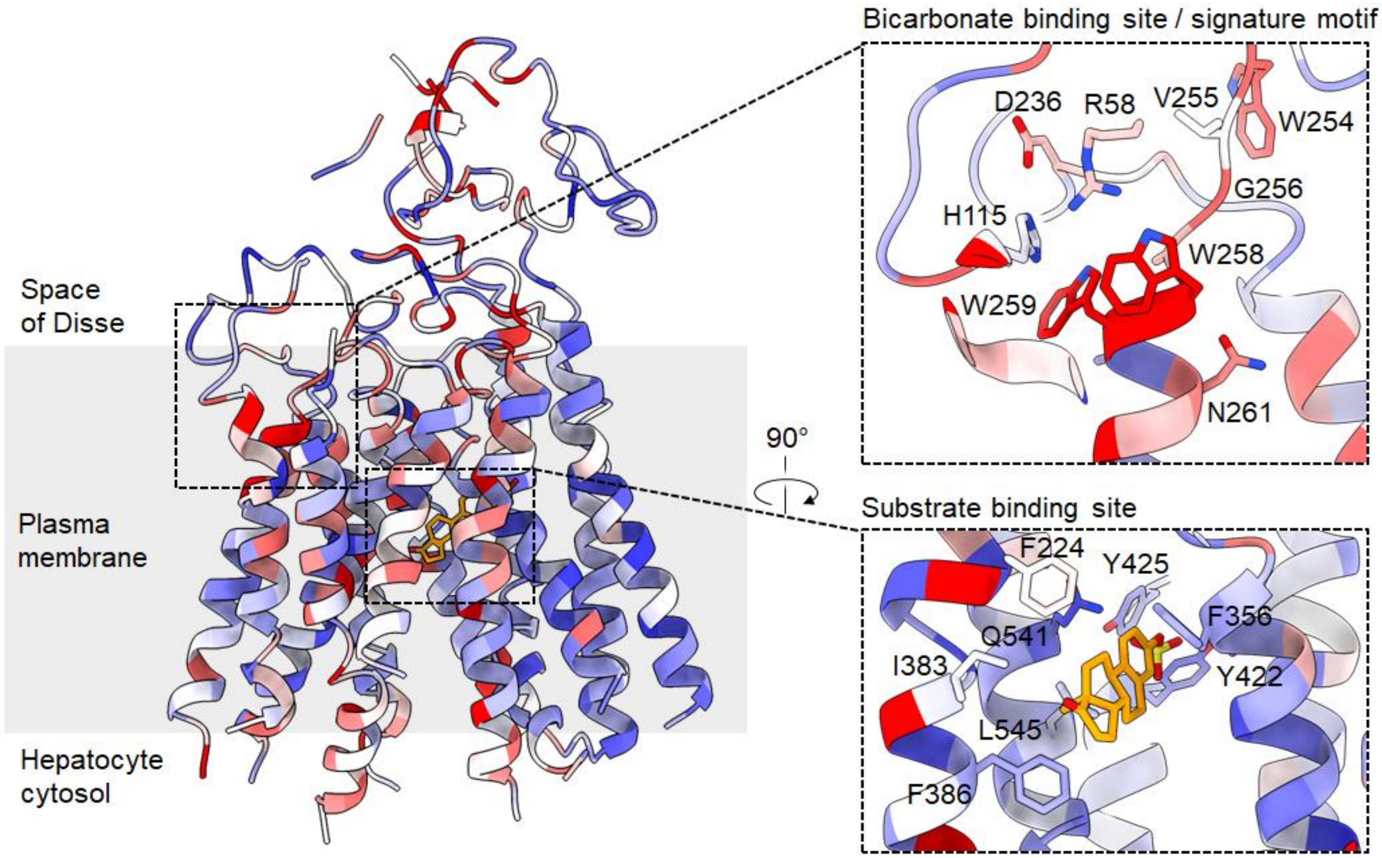

Supplementary Fig. 6. Sequence conservation in the family of human OATPs.
(*left*) Cartoon representation of E1S-bound structure of OATP1B1 colored based on conservation within human OATP family. Red and blue color indicate conserved and non-conserved residues, respectively. Eleven proteins in the OATP family was taken into analysis (those shown in Fig. 6b). (*right*) Close-up views on the bicarbonate binding site and the organic anion drug binding site. The relevant residues, which are described in the text, are displayed as sticks and labeled.

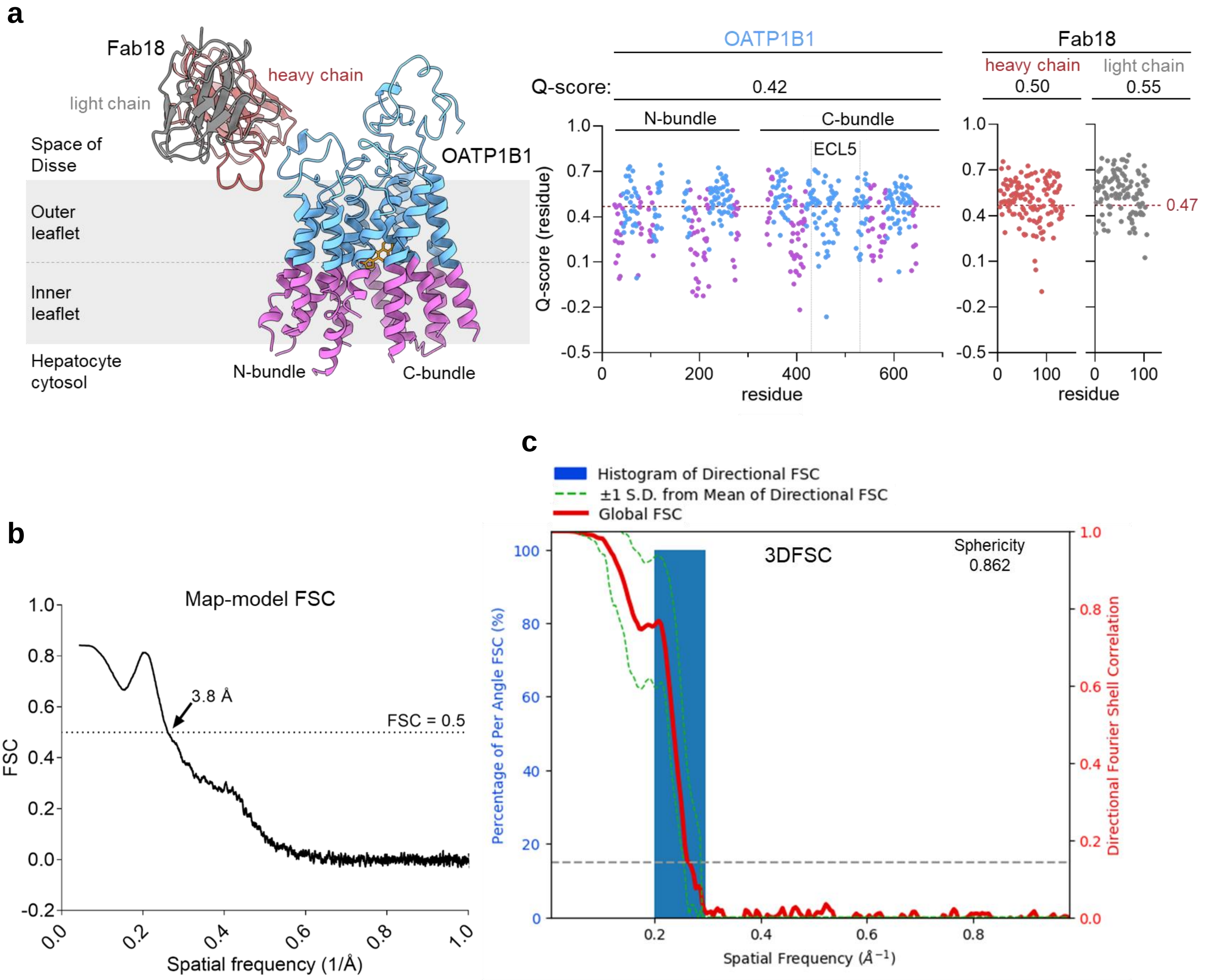

Supplementary Fig. 7. Analyses of EM density map and model of E1S-bound OATP1B1 in a complex with Fab18.
a Per-residue Q-scores for each residues. The colors of the ribbon representation on the left correspond to the Q-score data points on the right panel. The red, dotted line and the value indicates the expected Q-score based on the calculated resolution of the map (as per the 0.143 criterion). **b** The map-to-model FSC calculation. **c** Histogram and directional FSC generated on the remote 3DFSC processing server. Sphericity values are indicated.

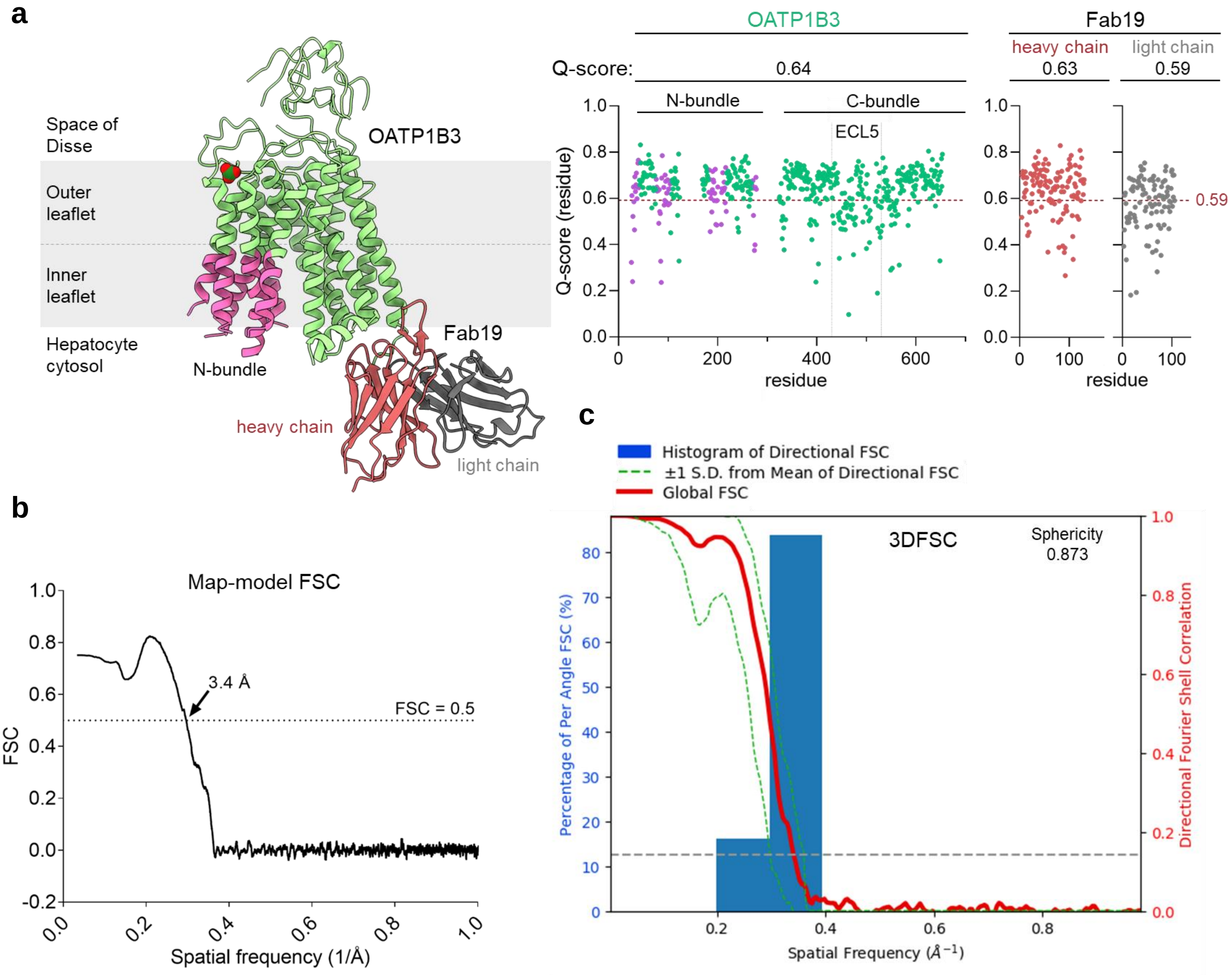

Supplementary Fig. 8. Analyses of EM density map and model of bicarbonate-bound OATP1B3 in a complex with Fab19.
a Per-residue Q-scores for each residues. The colors of the ribbon representation on the left correspond to the Q-score data points on the right panel. The red, dotted line and the value indicates the expected Q-score based on the calculated resolution of the map (as per the 0.143 criterion). **b** The map-to-model FSC calculation. **c** Histogram and directional FSC generated on the remote 3DFSC processing server. Sphericity values are indicated.

Supplementary Table 1. Cryo-EM data collection, refinement and validation statistics.

	**E1S-bound OATP1B1-Fab18** (EMDB-17677) (PDB **8PHW**)	**Bicarbonate-bound OATP1B3-Fab19** (EMDB-17655) (PDB **8PG0**)
**Data collection and processing**		
Magnification	165,000	165,000
Voltage (kV)	300	300
Electron exposure (e–/Å^2)	60.5	60.5
Defocus range (μm)	-0.6 – -2.2	-0.6 – -2.2
Pixel size (Å)	0.51	0.51
Super-resolution (Å)	0.255	0.255
Symmetry imposed	C1	C1
Movies (no.)	21,416	25,887
Initial particle images (no.)	4,375,486	5,947,675
Final particle images (no.)	104,547	75,610
Map resolution (Å)	3.55 / 3.67	2.97
FSC threshold	0.143	0.143
**Refinement**		
Initial model used (PDB code)	-	-
Model resolution (Å)	2.1	2.8
FSC threshold	0.143	0.143
Map sharpening *B* factor (Å^2)	-80	-70
Model composition		
Non-hydrogen atoms	5351	5812
Protein residues	689	737
Ligands	FY5: 1	BCT: 1 NAG: 1
Cholesterol	Y01: 1	CLR: 3
B factors (Å^2)		
Protein	85.63	39.75
Ligand	54.10	71.77
R.m.s. deviations		
Bond lengths (Å)	0.004	0.022
Bond angles (°)	0.708	0.951
Validation		
MolProbity score	2.62	2.04
Clashscore	10.67	16.45
Poor rotamers (%)	6.22	0.80
Ramachandran plot		
Favored (%)	92.53	95.37
Allowed (%)	7.47	4.21
Disallowed (%)	0.00	0.42
