# Supplementary material for: Structure of human drug transporters OATP1B1 and OATP1B3
Source: Nat Commun. 2023 Sep 18;14:5774. doi: 10.1038/s41467-023-41552-8 (PMC10507018; doi:10.1038/s41467-023-41552-8)
Supplement: Supplementary file 3 — Reporting Summary [file 41467_2023_41552_MOESM3_ESM.pdf]

nature portfolio

Corresponding author(s): Kaspar P. Locher

Last updated by author(s): Aug 30, 2023

Reporting Summary

Nature Portfolio wishes to improve the reproducibility of the work that we publish. This form provides structure for consistency and transparency in reporting. For further information on Nature Portfolio policies, see our Editorial Policies and the Editorial Policy Checklist.

Statistics

For all statistical analyses, confirm that the following items are present in the figure legend, table legend, main text, or Methods section.

n/a	Confirmed	
☐	☒	The exact sample size (n) for each experimental group/condition, given as a discrete number and unit of measurement
☐	☒	A statement on whether measurements were taken from distinct samples or whether the same sample was measured repeatedly
☐	☒	The statistical test(s) used AND whether they are one- or two-sided<br>*Only common tests should be described solely by name; describe more complex techniques in the Methods section.*
☒	☐	A description of all covariates tested
☐	☒	A description of any assumptions or corrections, such as tests of normality and adjustment for multiple comparisons
☐	☒	A full description of the statistical parameters including central tendency (e.g. means) or other basic estimates (e.g. regression coefficient) AND variation (e.g. standard deviation) or associated estimates of uncertainty (e.g. confidence intervals)
☐	☒	For null hypothesis testing, the test statistic (e.g. F, t, r) with confidence intervals, effect sizes, degrees of freedom and P value noted<br>*Give P values as exact values whenever suitable.*
☒	☐	For Bayesian analysis, information on the choice of priors and Markov chain Monte Carlo settings
☒	☐	For hierarchical and complex designs, identification of the appropriate level for tests and full reporting of outcomes
☒	☐	Estimates of effect sizes (e.g. Cohen's d, Pearson's r), indicating how they were calculated

Our web collection on statistics for biologists contains articles on many of the points above.

Software and code

Policy information about availability of computer code

Data collection	EPU2 software (Thermo Fisher Scientific)
Data analysis	Cryo-EM data processing: Relion 4.0, MotionCor2, Gctf_v1.06, CryoSPARC4.1,<br>Model building: Coot 0.9.8.3 (ccp4), PHENIX 1.20.1-4487,<br>Data analysis and visualization: UCSF ChimeraX 1.3, GraphPad Prism 9, ClustalOmega (online) and JalView2.11.2.6, SWISS-MODEL (online),<br>AutoDock Vina 1.2.0, DeepEMhancer, The PyMOL Molecular Graphics System v2.0 Schrödinger,

For manuscripts utilizing custom algorithms or software that are central to the research but not yet described in published literature, software must be made available to editors and reviewers. We strongly encourage code deposition in a community repository (e.g. GitHub). See the Nature Portfolio guidelines for submitting code & software for further information.

Data

Policy information about availability of data

All manuscripts must include a data availability statement. This statement should provide the following information, where applicable:

- Accession codes, unique identifiers, or web links for publicly available datasets
- A description of any restrictions on data availability
- For clinical datasets or third party data, please ensure that the statement adheres to our policy

Atomic coordinates of the E1S-bound OATP1B1-Fab18 and bicarbonate-bound OATP1B3-Fab19 models have been deposited in The Protein Data Bank (PDB) under

accession numbers 8PHW (https://doi.org/10.2210/pdb8PHW/pdb), 8PG0 (https://doi.org/10.2210/pdb8PG0/pdb). The three-dimensional cryo-EM density post-processed, masked maps have been deposited in the Electron Microscopy Data Bank (EMDB) under accession number 17677 (https://www.ebi.ac.uk/emdb/EMD-17677) and 17655 (https://www.ebi.ac.uk/emdb/EMD-17655).
The chemical structures of the compounds were taken from the Zinc15 database (https://zinc15.docking.org/) or PubChem (https://pubchem.ncbi.nlm.nih.gov/). Chemical structures were prepared using ChemDraw (Revvity Signals Software).

Research involving human participants, their data, or biological material

Policy information about studies with human participants or human data. See also policy information about sex, gender (identity/presentation), and sexual orientation and race, ethnicity and racism.

Reporting on sex and gender	n/a
Reporting on race, ethnicity, or other socially relevant groupings	n/a
Population characteristics	n/a
Recruitment	n/a
Ethics oversight	n/a

Note that full information on the approval of the study protocol must also be provided in the manuscript.

Field-specific reporting

Please select the one below that is the best fit for your research. If you are not sure, read the appropriate sections before making your selection.

☒ Life sciences ☐ Behavioural & social sciences ☐ Ecological, evolutionary & environmental sciences

For a reference copy of the document with all sections, see nature.com/documents/nr-reporting-summary-flat.pdf

Life sciences study design

All studies must disclose on these points even when the disclosure is negative.

Sample size	cell-based transport experiment was performed with N=4, 5 or 6 biological replicates
Data exclusions	no data was excluded
Replication	replication is part of the single-particle cryoEM data processing, whereas cell-based transport experiment is reproducible (performed multiple times, successful replication)
Randomization	n/a for structural study and cell-based transport experiment
Blinding	n/a for structural study and cell-based transport experiment

Reporting for specific materials, systems and methods

We require information from authors about some types of materials, experimental systems and methods used in many studies. Here, indicate whether each material, system or method listed is relevant to your study. If you are not sure if a list item applies to your research, read the appropriate section before selecting a response.

Materials & experimental systems

n/a	Involved in the study	
☐	☒	Antibodies
☐	☒	Eukaryotic cell lines
☒	☐	Palaeontology and archaeology
☒	☐	Animals and other organisms
☒	☐	Clinical data
☒	☐	Dual use research of concern
☒	☐	Plants

Methods

n/a	Involved in the study	
☒	☐	ChIP-seq
☒	☐	Flow cytometry
☒	☐	MRI-based neuroimaging

Antibodies

Antibodies used	produced in-house from the synthetic library: Fab18 and Fab19 (described in the text)
Validation	We described biophysical and biochemical characterization of the antibodies generated and used in this study.

Eukaryotic cell lines

Policy information about cell lines and Sex and Gender in Research

Cell line source(s)	Only Flp-In™ T-REx™ 293 Cell Line has been used in the study
Authentication	No authentication was performed.
Mycoplasma contamination	No testing for Mycoplasma contamination was performed.
Commonly misidentified lines (See ICLAC register)	No commonly misidentified cell lines were used.
